# Adding phosphorylation events to the core oscillator driving the cell cycle of fission yeast

**DOI:** 10.1371/journal.pone.0208515

**Published:** 2018-12-04

**Authors:** Dania Humaidan, Frank Breinig, Volkhard Helms

**Affiliations:** 1 Center for Bioinformatics, Saarland University, Saarbruecken, Germany; 2 Molecular and Cell Biology and Center of Human and Molecular Biology, Saarland University, Saarbruecken, Germany; King’s College London, UNITED KINGDOM

## Abstract

Much is known about the regulatory elements controlling the cell cycle in fission yeast (Schizosaccharomyces pombe). This regulation is mainly done by the (cyclin-dependent kinase/cyclin) complex (Cdc2/Cdc13) that activates specific target genes and proteins via phosphorylation events during the cell cycle in a time-dependent manner. However, more work is still needed to complement the existing gaps in the current fission yeast gene regulatory network to be able to overcome abnormalities in its growth, repair and development, i.e. explain many phenomena including mitotic catastrophe. In this work we complement the previously presented core oscillator of the cell cycle of fission yeast by selected phosphorylation events and study their effects on the temporal evolution of the core oscillator based Boolean network. Thereby, we attempt to establish a regulatory link between the autonomous cell cycle oscillator and the remainder of the cell. We suggest the unclear yet regulatory effect of phosphorylation on the added components, and discuss many unreported points regarding the temporal evolution of the cell cycle and its components. To better visualize the results regardless of the programming background we developed an Android application that can be used to run the core and extended model of the fission yeast cell cycle step by step.

## Introduction

The fission yeast is an intensely-studied model organism [1]. Although no fully connected gene regulatory network of the fission yeast has been presented to date, many aspects of its life cycle have been revealed [2]. Basically, the fission yeast goes through two types of differentiation: 1) Cellular proliferation that is a very essential function of the cell that drives the cell growth and development. Starting from one cell, this process ends with two identical cells. The four phases of the cell division cycle are as usual: G1—S—G2—M. 2) Sexual differentiation when there is lack of nutrients. This results in ascospores that can give cells if the nutrients are resupplied [3] and the cells go through the cellular proliferation. In this work, we will focus on the cell cycle of fission yeast, although we will discuss some points that are relevant to sexual differentiation as well.

To maintain cell development and avoid mistakes that may result in cell death or lead to abnormal cells such as tumors in the case of human, the eukaryotic cell division cycle is well controlled by two sorts of components termed cyclins, and cyclin dependent kinases (CDKs) [4]. CDKs form protein complexes with different cyclins and the changes in their activities result from the fact that the expression levels of the respective genes oscillate during the cell cycle. In the fission yeast, one CDK (Cdc2) is essential so that a minimal network with only this CDK and its cyclin (Cdc13) can drive the cell cycle in the same manner as the wild type [5] [6].

Phosphorylation has very important roles in biological processes such as signal transduction, gene expression and cell differentiation [7] as phosphorylation of protein residues may activate or deactivate enzymes and transcription factors. During the cell division cycle, many proteins are phosphorylated or dephosphorylated [8] and this is important to switch correctly between cycle phases. Phosphorylation events are also important control mechanism by which the core oscillator affects the remaining genes of the cell and thus drives the cell cycle. An important role of phosphorylation not only in fission yeast but in all eukaryotes is phosphorylation of the C-terminal domain of RNA polymerase II that has central roles in the integrated events of gene expression [9].

For fission yeast, Nurse and colleagues studied the phosphorylation events happening during the cell division cycle of the CDK-cyclin construct mentioned above. They found that this protein complex phosphorylates 275 substrates during the cell cycle, involving a total number of 149 distinct proteins. The phosphorylation phase varied between early (G1/S), mid (G2/M) or late (M) [8].

To describe and characterize the regulation of the cell cycle in mathematical terms, an ordinary differential equations model was presented [10] describing how the transitions are regulated. It consists of the main complex of cdc2/cdc13 and further proteins that either positively or negatively regulate it.

**Fig 1:**
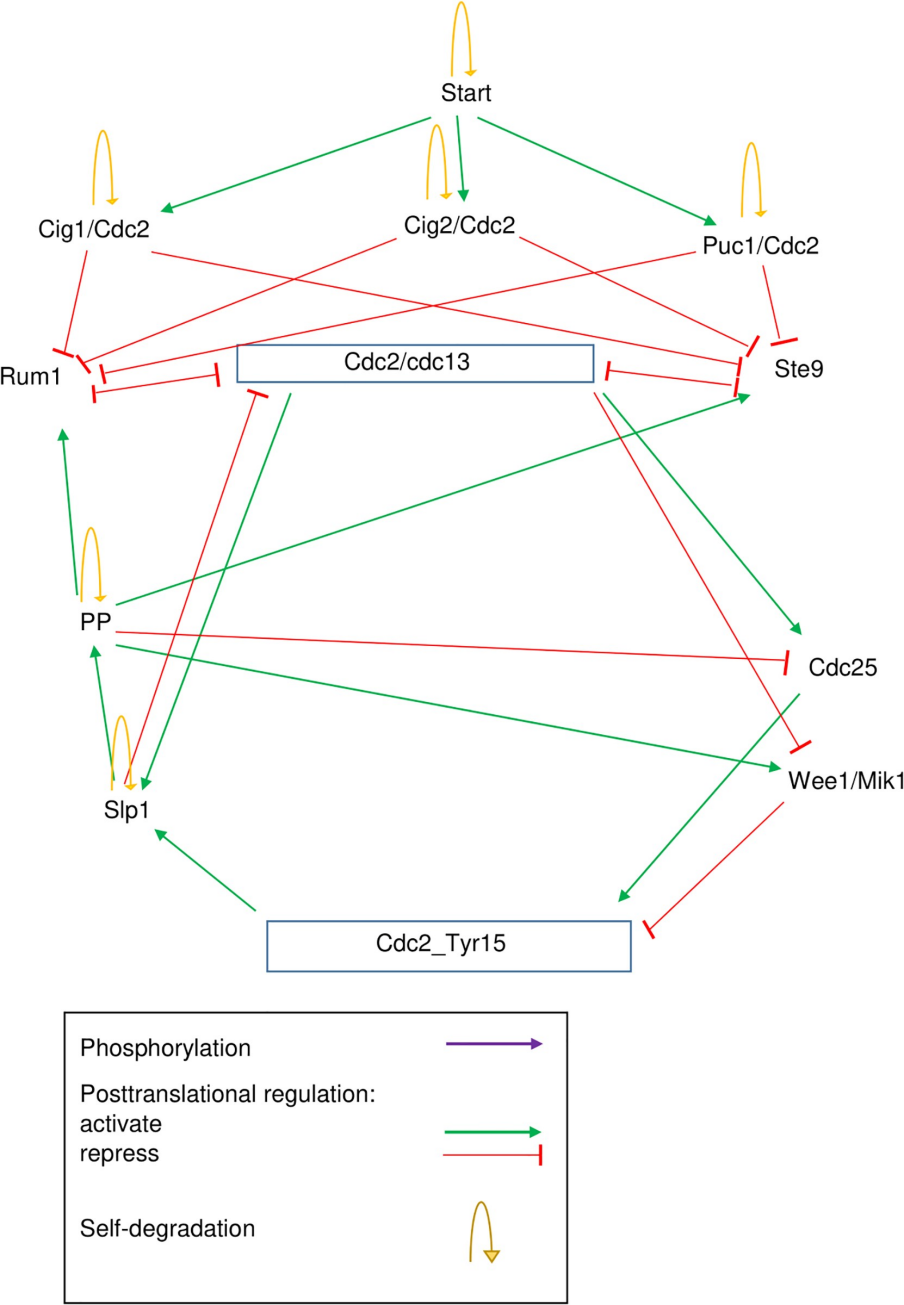
Boolean network model of fission yeast core oscillator by Davidich and Bornholdt (2008). It consists of the Cdc2/Cdc13 complex and its regulators. Solid arrows represent activating interactions, dashed arrows mark repressive interactions, and yellow arrows represent self-degradation loops, respectively. Figure adapted from [12].

A simpler Boolean network representation of the core oscillator of the cell cycle of fission yeast was presented by Davidich and Bornholdt [11] [12]. This model contains the main complex Cdc2/Cdc13, its positive regulators including the phosphatase Cdc25 and several protein complexes formed between Cdc2 and the remaining cyclins Cig1, Cig2 and Puc1 of fission yeast, and several transcription factors that may repress the expression of Cdc2 including Slp1, Rum1, Ste9 and the phosphatase PP. Cdc2 can also be regulated by phosphorylation of Tyr_15, which reduces its activity [11] [12]. Fig 1 shows the schema of this Boolean network and Table 1 shows its temporal evolution.

**Table 1:** The temporal evolution of the cell cycle core oscillator according to Davidich and Bornholdt Boolean network model (2013).

Time	Phase	Start	Cig1/Cdc2	Cig2/Cdc2	Puc1/Cdc	Cdc2/Cdc13	Ste9	Rum1	Slp1	Cdc2_Tyr15	Wee1/Mik1	Cdc25	PP
1	Start	1	0	0	0	0	1	1	0	0	1	0	0
2	G1	0	1	1	1	0	1	1	0	0	1	0	0
3	G1/S	0	0	0	0	0	0	0	0	0	1	0	0
4	G2	0	0	0	0	1	0	0	0	0	1	0	0
5	G2	0	0	0	0	1	0	0	0	0	0	1	0
6	G2/M	0	0	0	0	1	0	0	0	1	0	1	0
7	G2/M	0	0	0	0	1	0	0	1	1	0	1	0
8	M	0	0	0	0	0	0	0	1	1	0	1	1
9	M	0	0	0	0	0	1	1	0	1	1	0	1
10	G1	0	0	0	0	0	1	1	0	0	1	0	0

The table shows the binary states of genes representing the active (1) or inactive (0) state. Shown is also the phase of the cell cycle.

Although this network includes the core oscillator of the fission yeast cell cycle, it misses many phosphorylation events that happen during the cell cycle. Understanding these events and bridging them to the core oscillator is an important step towards the formation of a full gene regulatory network.

In this work, we present an extended model how the core oscillator of the cell cycle of fission yeast drives phosphorylation events in the cell and how these may couple back on the core oscillator. We discuss and suggest the possible scenarios and effects they might have on the behavior of cell cycle events. To do this, we start from the cell cycle core oscillator and add phosphorylation events detected by [8] that are driven by the main complex (Cdc2/Cdc13). In this manner, we derived a core network of phosphorylation events with genes that feed back onto the core oscillator genes. Then, we combine our network with the core oscillator and construct a Boolean network to run the final model. To enable understanding the results and the temporal evolution of the cell cycle regardless of the programming background, an Android application was developed that shows the steps of the cell cycle with the added phosphorylation events. Finally, we raise many open points that are still unclear and need more investigation regarding the genes related to the fission yeast cell cycle.

## 1 Methods

### 1.1 The fission yeast cell cycle control network

The fission yeast cell cycle is tightly regulated by a set of transcription factors and their target genes. Many genes have been identified to have specific roles during the cell cycle. We retrieved the roles of the considered genes from the online database for fission yeast PomBase [13] and complemented this by further information retrieved from the literature.

### 1.2 Phosphorylation events during the cell cycle

In order to study the effects of phosphorylation events on the core oscillator during the cell cycle, we checked which of the 275 phosphorylation substrates [8] of Cdc2 (representing 149 unique proteins) are themselves transcription factors. This gave 7 transcription factors according to PomBase [13], and 2 additional ones according to DBD [14]. Table 2 lists the transcription factors found among the phosphorylation substrates.

**Table 2:** Phosphorylation substrates of Cdc2 that are transcription factors themselves.

Gene name	Gene product
Sep1	forkhead transcription factor Sep1
Bqt4	bouquet formation protein Bqt4
Atf1	transcription factor, Atf-CREB family Atf1
Snt1	Set3 complex subunit Snt1
Fkh2	forkhead transcription factor Fkh2
Rst2	transcription factor Rst2
Hsr1	transcription factor Hsr1
Scr1	transcription factor Scr1
Cdc10	MBF transcription factor complex subunit Cdc10

The next step was to find the target genes of these transcription factors. Table 3 lists the known target genes of the 7 transcription factors according to PomBase [13] and Bushel et al. (2009) [2]. Note that some of the target genes have not been assigned a Gene Standard Name yet.

**Table 3:** Target genes of the phosphorylated transcription factors.

Gene name	Target genes
Sep1	Plo1, Fin1, Mid1, Spo12, Sig2, Ppb1, Cdc15, Mcm2, Myp2, Klp5, Rum1, Ark1
Atf1	Ntp1, Ecl1, Ctt1, Ish1, Cdc13, Gpx1, Agl1, Fbp1
Fkh2	Myp2, Plo1, Klp5, Rum1, Ark1, Ste11
Rst2	Fnp1, Ste11
Hsr1	SPAC26H5.07C, SPCC594.01, Bud32, Vtc4, SPBC725.03, SPCC613.03, SPBC83.16C, Urg1, Dsc5, Crs103
Scr1	Gld1
Cdc10	Ctp1, Cig2, Cdc18, Nrm1, Mik1, Cdt1

### 1.3 Gene expression data

According to [2] [15], around 2000 genes out of the ca. 5000 genes of wild-type fission yeast show oscillating expression levels during the cell cycle.

### 1.4 GO terms

We used the online tool DAVID [16] [17] for functional annotation (GO terms) of the oscillating target genes.

### 1.5 Boolean network and Android implementation

The Boolean network implemented in this work was first implemented as a simple Python code. To make the model more accessible to biologists, we then also implemented a version using the Android Studio IDE that can be run on a smart phone under the Android operating system. The application implements the original Davidich & Bornholdt model as well as the extended Boolean network presented in this work and also enables the user to change some options to check other possibilities that are discussed in this work.

The application was made up from five screens called (activities) with a total of around 1000 lines of code. The activities are the following:

- Main activity: provides an introduction to the cell cycle and its different phases.
- Introduction activity: shows the current cell cycle core oscillator network. Two buttons allow the user to process to the temporal evolution activities of the current and extended models.
- Temporal evolution activity: contains an interactive network of the current cell cycle core oscillator. If started with the run button there is visual information on which genes are turned on or off at the current phase of the cell cycle that is shown above the network and. A pause option can be activated at any phase.
- Extended temporal activity: this is similar to the previous activity but contains an edit option to go to the edit activity.
- Edit activity: allows the use to edit the phosphorylation effects of the three phosphorylation events added to the current model.

The application provides four functionalities (Use cases) as shown in the use case diagram in S1 Fig.

## 2 Results

### 2.1 Target genes whose expression oscillates during the cell cycle

Due to enzymatic activity of (Cdc2/Cdc13), the phosphorylation levels of Cdc2 substrates oscillate during the cell cycle. First, we analyzed whether the annotated target genes of the reported phosphorylation substrates [8] that are themselves transcription factors show periodic changes of expression during cell cycle. Among the 40 distinct target genes we extracted from Pombase [12] and [2], 28 genes oscillate during the cell cycle (70% of the genes). This reflects a 1.75-fold enrichment over the overall fraction of oscillating genes. S1 Table lists the target genes with their PomBase IDs and their oscillation ranks according to [2] and [15].

### 2.2 GO terms enrichment

We expected that the target genes with oscillating expression levels during the cell cycle may show GO term enrichment regarding functions related to the cell cycle. Using DAVID [16] [17], and providing the 40 target genes in Table 3 as input, we found that most oscillating target genes indeed have GO terms directly related to the cell cycle such as cell cycle (GO:0007049), cell division (GO:0051301) and cytoskeleton (GO:0005856). In contrast, the 12 non-oscillating target genes tend not to be involved in the cell cycle. Results are shown in Table 4.

**Table 4:** GO term statistics of the target genes.

GO term	of 40 Target Genes	of TGs of 4 TFs	of 28 oscillating TGs	of 12 non oscillating TGs	p-value	FDR
Cell cycle	14	14	12	2	1.12E-07	1.19E-04
Cytoskeleton	9	9	8	1	3.39E-05	0.0359912
Cell division	12	12	11	1	3.78E-07	4.01E-04
Mitotic spindle pole body	6	6	6	0	0.0197135	18.521663
Mitotic spindle midzone	4	4	4	0	6.14E-04	0.6293664
Mitosis	12	12	11	1	7.73E-09	8.20E-06
Kinase	6	5	5	1	0.0102281	10.32913

### 2.3 Adding phosphorylation events

In order to study the effect of phosphorylation events on the cell cycle core oscillator, we aimed at extending the core network, that is centered on the Cdc2/Cdc13 complex by those transcription factors whose target genes feed back onto either Cdc2 or Cdc13 or both. We added the following regulatory links:

- Cdc2/Cdc13: phosphorylates Cdc10, Atf1 during G2/M, Sep1 during G2/M and Fkh2 during G1/S [9]. Cdc2/Cdc13 peaks at M and is inhibited by Rum1 [18].
- Sep1: activates Rum1 during M phase [13].
- Fkh2: activates Rum1 during M phase [13].
- Atf1: activates the expression of Cdc13 promoting the G2/M transition [19].
- Rum1: Binds to Cdc2/Cdc13 complex and promotes Cdc13 proteolysis [18]. Cdc2-cyclin complexes target Rum1 for degradation [20].
- Cig2: depends on Cdc10 [21].
- Mik1: is regulated by Cdc10 [13], is active during G1/S [22].
- Cdc10: part of the MBF (Mlu1 cell cycle box Binding Factor complex), has a constant level through cell cycle [23].

The given citations point to the original experimental papers where this regulatory effect was reported.

**Fig 2:**
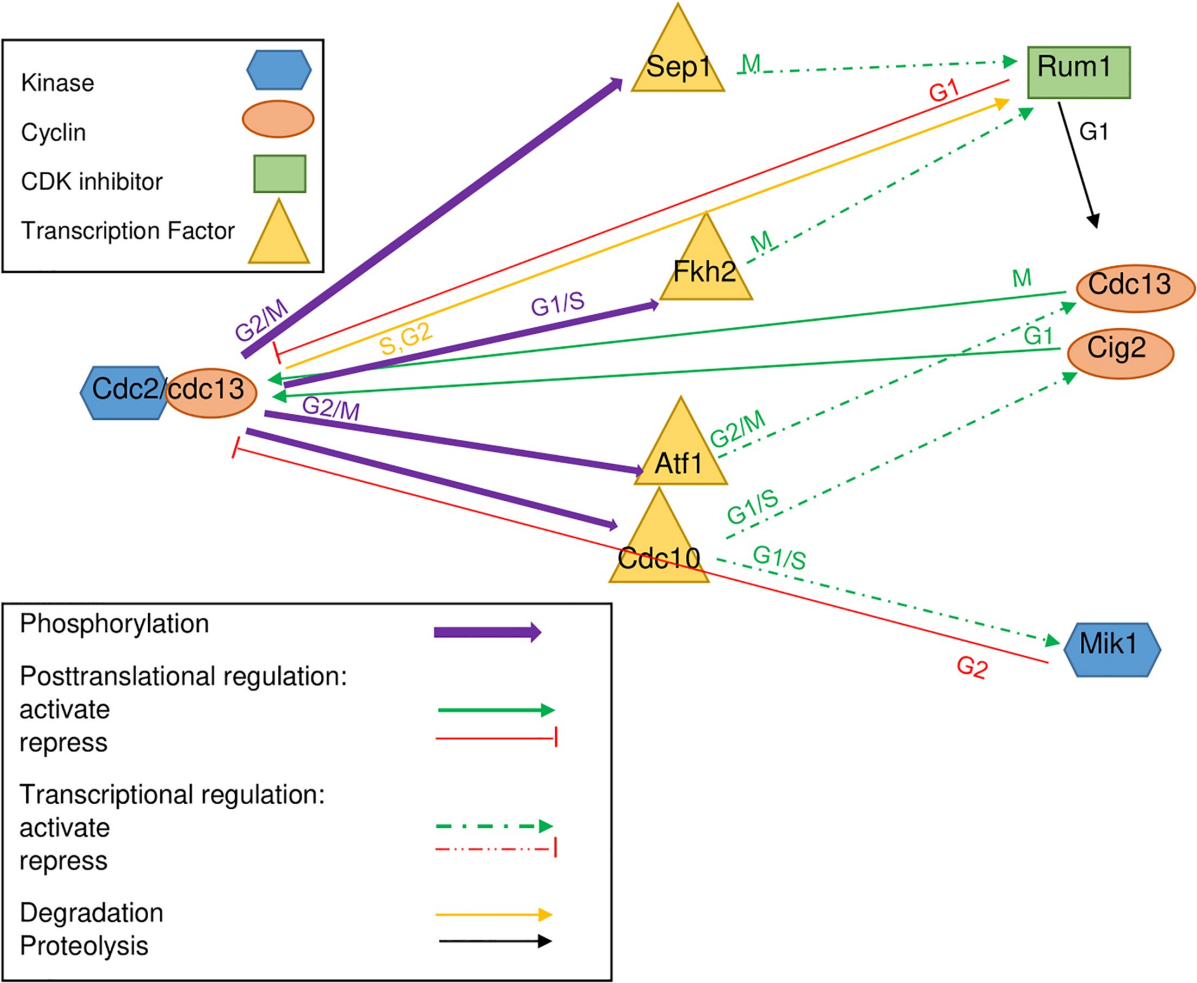
The core network of events involving Cdc2/Cdc13, the selected substrates and their target genes. Shown are phosphorylation and regulation events involving the Cdc2/Cdc13 complex and the four selected substrates (transcription factors—yellow triangles) with their target genes that feed back onto Cdc2/Cdc13.

Fig 2 illustrates these additional processes together with the phase of the cell cycle when they were reported to occur.

### 2.4 The extended BN model

**Fig 3:**
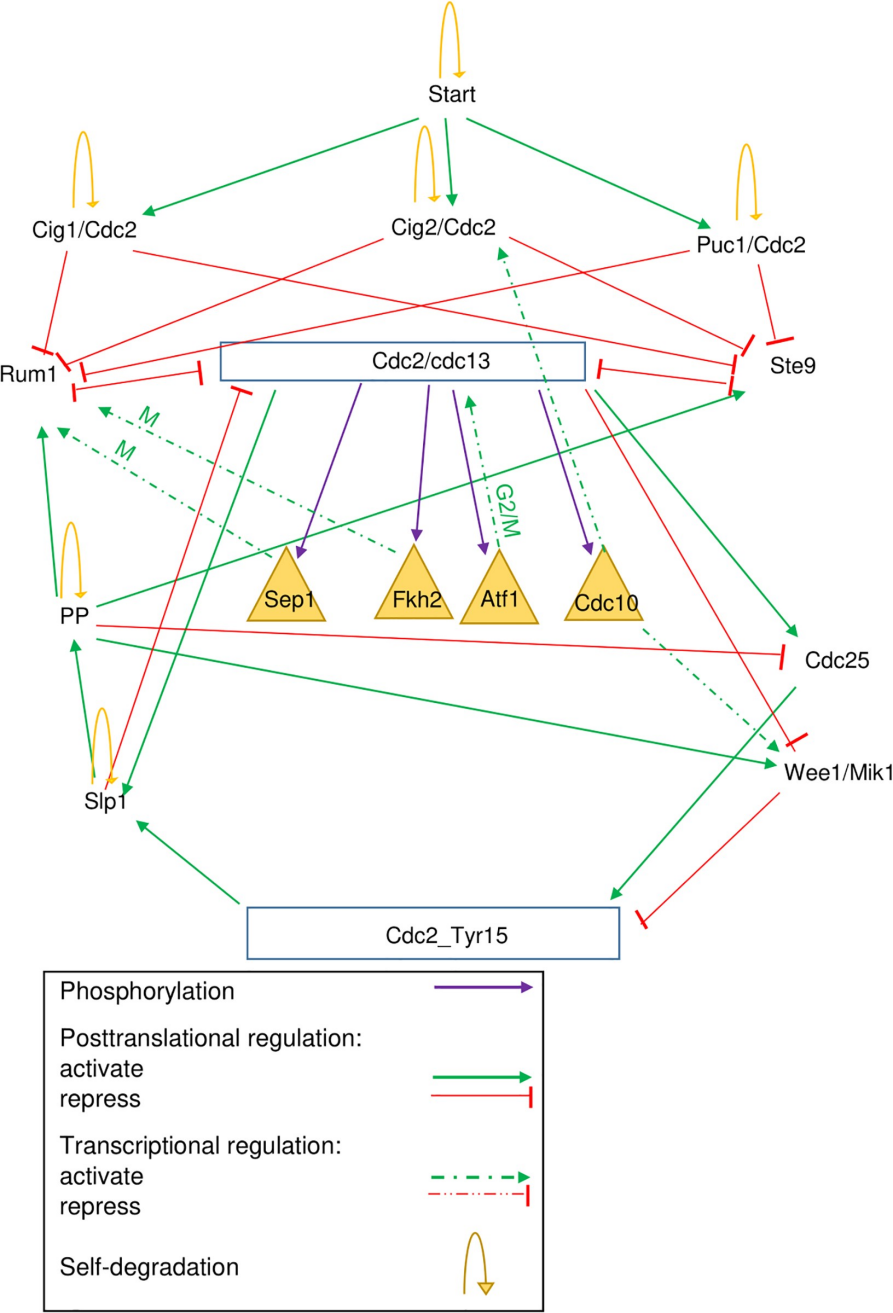
The extended model of the core oscillator with added phosphorylation events.

Next, we extended the original Boolean Network model by Davidich and Bornholdt of the fission yeast cell cycle [11] [12] by the events described in Fig 2. Fig 3 shows the extended model.

The four phosphorylation substrates seem to have a positive regulatory effect on the components of the core oscillator of the cell cycle.

We implemented the Boolean network of the core oscillator and then added the new nodes to the network structure. In order to run the network, we added some rules that ensure that the added nodes are only effective during the specific phase of the cell cycle as reported in the literature.

Concerning the two components of the Cdc2/Cdc13 complex, it is known that Cdc13 is always synthesized. However, at the same time many regulators lead to its degradation so that its level oscillates during the cell cycle [12]. On the other hand, the level of Cdc2 is quite constant during the cell cycle [24].

Atf1 activates the expression of Cdc13 [19] during G2/M which is when Cdc2/Cdc13 reaches high activity and the cell enters mitosis. Therefore, so we added a positive effect from Atf1 on Cdc2/Cdc13 during G2/M phase.

Fkh2 and Sep1 both activate Rum1 during M phase [13], though it has been suggested that Fkh2 has a negative effect on the Sep1 dependent genes [25]. In the model, Rum1 should be inhibited during G1/S which is done by the Start Kinases (Cig1/Cdc2, Cig2/Cdc2 and Puc1/Cdc2). Therefore, we added the activation effect by Fkh2 and Sep1 during M phase.

Cdc10 remains phosphorylated throughout the cell cycle with no changes in its level [24].

Based on these considerations, we implemented a Boolean network. The results showed that almost the same oscillation takes place. The only difference from the original DB model is that Rum1 stays active for one more time step (marked in red) during M phase, as it is being activated by Sep1 and Fkh2. The results of the core oscillator are shown in Table 5 and the results of the final model are shown in Table 6.

**Table 5:** Running the core oscillator Boolean network.

Time	Start	Cig1/Cdc2	Cig2/Cdc2	Puc1/Cdc2	Cdc2/Cdc13	Ste9	Rum1	Slp1	Cdc2_Tyr15	Wee1/Mik1	Cdc25	PP
1	1	0	0	0	0	1	1	0	0	1	0	0
2	0	1	1	1	0	1	1	0	0	1	0	0
3	0	0	0	0	0	0	0	0	0	1	0	0
4	0	0	0	0	1	0	0	0	0	1	0	0
5	0	0	0	0	1	0	0	0	0	0	1	0
6	0	0	0	0	1	0	0	0	1	0	1	0
7	0	0	0	0	1	0	0	1	1	0	1	0
8	0	0	0	0	0	0	0	1	1	0	1	1
9	0	0	0	0	0	1	1	0	1	1	0	1
10	0	0	0	0	0	1	1	0	0	1	0	0
11	0	0	0	0	0	1	1	0	0	1	0	0
12	0	0	0	0	0	1	1	0	0	1	0	0
13	0	0	0	0	0	1	1	0	0	1	0	0
14	0	0	0	0	0	1	1	0	0	1	0	0
15	0	0	0	0	0	1	1	0	0	1	0	0
16	0	0	0	0	0	1	1	0	0	1	0	0
17	0	0	0	0	0	1	1	0	0	1	0	0
18	0	0	0	0	0	1	1	0	0	1	0	0
19	0	0	0	0	0	1	1	0	0	1	0	0

**Table 6:** Running the final model Boolean network.

Time	Start	Cig1/Cdc2	Cig2/Cdc2	Puc1/Cdc2	Cdc2/Cdc13	Ste9	Rum1	Slp1	Cdc2_Tyr15	Wee1/Mik1	Cdc25	PP
1	1	0	0	0	0	1	1	0	0	1	0	0
2	0	1	1	1	0	1	1	0	0	1	0	0
3	0	0	0	0	0	0	0	0	0	1	0	0
4	0	0	0	0	1	0	0	0	0	1	0	0
5	0	0	0	0	1	0	0	0	0	0	1	0
6	0	0	0	0	1	0	0	0	1	0	1	0
7	0	0	0	0	1	0	0	1	1	0	1	0
8	0	0	0	0	0	0	***1***	1	1	0	1	1
9	0	0	0	0	0	1	1	0	1	1	0	1
10	0	0	0	0	0	1	1	0	0	1	0	0
11	0	0	0	0	0	1	1	0	0	1	0	0
12	0	0	0	0	0	1	1	0	0	1	0	0
13	0	0	0	0	0	1	1	0	0	1	0	0
14	0	0	0	0	0	1	1	0	0	1	0	0
15	0	0	0	0	0	1	1	0	0	1	0	0
16	0	0	0	0	0	1	1	0	0	1	0	0
17	0	0	0	0	0	1	1	0	0	1	0	0
18	0	0	0	0	0	1	1	0	0	1	0	0
19	0	0	0	0	0	1	1	0	0	1	0	0

The entry marked in bold and italic denotes the only difference between this final model and the current model in Table 5.

### 2.5 Android application

The android application starts with a simple introductory page about the cell cycle, showing the phases with an illustration of the cell in each phase.

On the next page, one can check the current core oscillator of the cell cycle of fission yeast according to Davidich and Bornholdt [12] represented as a network of genes and the interactions between them. From there, two options are available: either to run the current Boolean Network model, or to check the extended model, including the phosphorylation events added in this work.

If the user chooses to check the current model of the fission yeast cell cycle, then the network is shown with a state indicator that is turned green when the gene is active and turned off otherwise. Using the “Run” button, the Boolean Network simulation of the cell cycle is started and the genes’ states start to change as the state of each gene is calculated at each time step. If the user wished to pause the running for a while to check the genes’ states during a specific phase, this can be done using the “Pause” switch. After turning the “Pause” switch off, the cell cycle continues.

The other option is to select the extended model that is the DB model with the phosphorylation events added in this work. In addition to the run and pause options, the user can also edit the phosphorylation effect of the Cdc2/Cdc13 complex on the three substrates Sep1, Atf1 and Fkh2 between (Activation) or (Inhibition). Using the “Edit” button the user can switch the phosphorylation effects and the edits will then be applied to the model including the network reactions and the rules used to calculate the states after each time step.

The final application can be downloaded from the public repository: https://github.com/Dashbioinfo/FissionYeastCellCycle. A short video tutorial on how to use it can be found in S1 File.

## 3 Discussion

We have extended the current cell cycle core oscillator of fission yeast by several phosphorylation events based on recently reported experimental evidence. We presented a model for the regulatory logic of some genes, whose products are being phosphorylated during the cell cycle of fission yeast, on the remaining genes of the core oscillator of the cell cycle. Although the available knowledge could be integrated in a consistent manner, this work also revealed critical gaps in existing knowledge of the genes related to the cell cycle of fission yeast. Most importantly, it is unknown how the activation levels are positively or negatively regulated by phosphorylation. On the other hand, since no full gene regulatory network of fission yeast has been presented so far, not all target genes and regulatory roles are known. An example are the genes Fkh2 and Sep1 that both activate Rum1 during M phase [13]. However, it has been reported that Fkh2 has a negative effect on Sep1 targets [25], so further information about how they function together is needed. Another example is the gene Cdc10. Its protein product is phosphorylated during the cell cycle and this is required for its function in starting the cell cycle of fission yeast. However, Cdc10 shows no steady-state changes in level, so its job is not regulated at the transcription level [23], and since it is phosphorylated throughout the entire cell cycle, this raises the demand for more details on how it starts the cell cycle.

To identify the genes whose expression levels oscillate during the cell cycle of fission yeast, we used the gene expression data from wild-type, as this information was not available for the specific strain of single cyclin-CDK used by Nurse and his colleagues on phosphorylation events during the cell cycle of fission yeast [8] and Gérard and his colleagues on cell cycle control by a minimal Cdk network [5], and because this minimal network can control DNA synthesis and mitosis in the same manner as the wild-type [5]. We limited our analysis to the transcription factors among the Cdc2 phosphorylation substrates to follow the further effects that then feed back onto Cdc2. We have also checked for kinases among the substrates to check whether they later on phosphorylate transcription factors of the cell cycle core oscillator but did not find information that is worth mentioning.

Besides activating transcription factors via phosphorylation, also their translocation to the nucleus, ligand binding or endoproteolytic activation may play a role. Furthermore, activation of transcription factors via phosphorylation may not be a on/off event, but may require that certain functional thresholds are exceeded.

Then we implemented the extended model as a Boolean network to check the temporal evolution of the model All of these effects could, in principle, be incorporated into Boolean Networks and ODE models, but their parametrization necessitates additional experimental evidence.

We then suggested possible effects of phosphorylation events on relevant genes products as well as a possible mechanism to restart a new cell cycle. Note that these effects have not yet been experimentally shown:

1. Sep1 is phosphorylated during G2/M [8] and it should be active during M phase so that it is able to activate Rum1 [13]. If nothing else is activating Sep1 during M phase, then one may speculate that its phosphorylation might lead to its activation.
2. Fkh2 is phosphorylated during G1/S phase [8] and this phosphorylation is important to suppress the expression of Ste11 and proceed to mitotic cell cycle [26]. Since Fkh2 activates Ste11 transcriptionally [13], then its phosphorylation might lead to its deactivation and then inhibition of Ste11 expression so that the cell can proceed with the cell cycle. Additionally, Fkh2 should also be active during M phase so that it is able to activate Rum1 [13]. Further information is needed to determine if it stays active by dephosphorylation until its turn during M phase or if another factor is responsible for its activity.
3. Atf1 is phosphorylated during G2/M phase [8] and it activates the expression of Cdc13 promoting G2/M transition [19]. Thus, the phosphorylation might have a causal activation role in this case. One more thing to mention about Atf1 is that it is dephosphorylated twice [8]. On the one hand, Atf1 has a role in switching to sexual differentiation, by being phosphorylated by Spc1 [27]. On the other hand, it is phosphorylated during G2/M phase [8]. We can speculate that the first phosphorylation happens during G1 if the cell decides to switch to sexual differentiation, and then the dephosphorylation happens during G1/S [8], and that the second phosphorylation happens during G2/M if the cells decides to go through the mitotic cell cycle, then it is dephosphorylated at mitotic exit.

These effects were implemented and the temporal evolution is shown in Table 7.

**Table 7:** Running the extended model Boolean network with phosphorylation effects.

Time	Start	Cig1/Cdc2	Cig2/Cdc2	Puc1/Cdc	Cdc2/Cdc13	Ste9	Rum1	Slp1	Cdc2_Tyr15	Wee1/Mik1	Cdc25	PP	Sep1	Fkh2	Atf1	Cdc10
1	1	0	0	0	0	1	1	0	0	1	0	0	0	1	0	1
2	0	1	1	1	0	1	1	0	0	1	0	0	0	1	0	1
3	0	0	0	0	0	0	0	0	0	1	0	0	0	0	0	1
4	0	0	0	0	1	0	0	0	0	1	0	0	0	0	0	1
5	0	0	0	0	1	0	0	0	0	0	1	0	0	0	0	1
6	0	0	0	0	1	0	0	0	1	0	1	0	1	0	1	1
7	0	0	0	0	1	0	0	1	1	0	1	0	1	0	1	1
8	0	0	0	0	0	0	1	1	1	0	1	1	1	0	1	1
9	0	0	0	0	0	1	1	0	1	1	0	1	1	0	1	1
10	0	0	0	0	0	1	1	0	0	1	0	0	1	0	1	1
11	0	0	0	0	0	1	1	0	0	1	0	0	1	0	1	1
12	0	0	0	0	0	1	1	0	0	1	0	0	1	0	1	1
13	0	0	0	0	0	1	1	0	0	1	0	0	1	0	1	1
14	0	0	0	0	0	1	1	0	0	1	0	0	1	0	1	1
15	0	0	0	0	0	1	1	0	0	1	0	0	1	0	1	1
16	0	0	0	0	0	1	1	0	0	1	0	0	1	0	1	1
17	0	0	0	0	0	1	1	0	0	1	0	0	1	0	1	1
18	0	0	0	0	0	1	1	0	0	1	0	0	1	0	1	1
19	0	0	0	0	0	1	1	0	0	1	0	0	1	0	1	1

It has been proposed that the main components of the cell cycle core oscillator including Rum1, Ste9, Cdc25, Wee1 and Mik1 are dephosphorylated at the end of the cycle [28]. On the other hand, all the substrates that are phosphorylated by Cdc2/Cdc13 -including early, middle and late substrates- are dephosphorylated at mitotic exit [8]. Additionally, according to [8], Fkh2 is being dephosphorylated during S phase.

We added these effects to three of the substrates we added: Sep1, Fhk2 and Atf1. The temporal evolution is shown in Table 8.

**Table 8:** Running the extended model Boolean network with phosphorylation effects and dephosphorylation at mitotic exit.

Time	Start	Cig1/Cdc2	Cig2/Cdc2	Puc1/Cdc	Cdc2/Cdc13	Ste9	Rum1	Slp1	Cdc2_Tyr15	Wee1/Mik1	Cdc25	PP	Sep1	Fkh2	Atf1	Cdc10
1	1	0	0	0	0	1	1	0	0	1	0	0	0	1	0	1
2	0	1	1	1	0	1	1	0	0	1	0	0	0	1	0	1
3	0	0	0	0	0	0	0	0	0	1	0	0	0	0	0	1
4	0	0	0	0	1	0	0	0	0	1	0	0	0	0	0	1
5	0	0	0	0	1	0	0	0	0	0	1	0	0	1	0	1
6	0	0	0	0	1	0	0	0	1	0	1	0	1	1	1	1
7	0	0	0	0	1	0	0	1	1	0	1	0	1	1	1	1
8	0	0	0	0	0	0	1	1	1	0	1	1	1	1	1	1
9	0	0	0	0	0	1	1	0	1	1	0	1	1	1	1	1
10	0	0	0	0	0	1	1	0	0	1	0	0	1	1	1	1
11	0	0	0	0	0	1	1	0	0	1	0	0	0	1	0	1
12	0	0	0	0	0	1	1	0	0	1	0	0	0	1	0	1
13	0	0	0	0	0	1	1	0	0	1	0	0	0	1	0	1
14	0	0	0	0	0	1	1	0	0	1	0	0	0	1	0	1
15	0	0	0	0	0	1	1	0	0	1	0	0	0	1	0	1
16	0	0	0	0	0	1	1	0	0	1	0	0	0	1	0	1
17	0	0	0	0	0	1	1	0	0	1	0	0	0	1	0	1
18	0	0	0	0	0	1	1	0	0	1	0	0	0	1	0	1
19	0	0	0	0	0	1	1	0	0	1	0	0	0	1	0	1

In addition, the following case can be discussed: The cell cycle starts when the START, Ste9, Wee1 and Rum1 components are on, but goes back to this start state with the START element turned off. This questions the idea of having a way to start a new run of the cell cycle again. It has been reported that the cell cycle is started by the Res1p-Cdc10p complex that is promoted by many ways including the phosphorylation of Cdc10 by Cdc2 [29], and among the added phosphorylation substrates, the Cig2 appearance was reported to depend on Cdc10 [21]. One possible scenario we can suggest is that the Cdc10 activates Cig2 indirectly by activating the START component, thus starting a new run of the cell cycle. Temporal evolution is shown in Table 9.

**Table 9:** Running the extended model Boolean network with phosphorylation effects, dephosphorylation at mitotic exit and cycle restart by cdc10.

Time	Start	Cig1/Cdc2	Cig2/Cdc2	Puc1/Cdc	Cdc2/Cdc13	Ste9	Rum1	Slp1	Cdc2_Tyr15	Wee1/Mik1	Cdc25	PP	Sep1	Fkh2	Atf1	Cdc10
1	1	0	0	0	0	1	1	0	0	1	0	0	0	1	0	1
2	0	1	1	1	0	1	1	0	0	1	0	0	0	1	0	1
3	0	0	0	0	0	0	0	0	0	1	0	0	0	0	0	1
4	0	0	0	0	1	0	0	0	0	1	0	0	0	0	0	1
5	0	0	0	0	1	0	0	0	0	0	1	0	0	1	0	1
6	0	0	0	0	1	0	0	0	1	0	1	0	1	1	1	1
7	0	0	0	0	1	0	0	1	1	0	1	0	1	1	1	1
8	0	0	0	0	0	0	1	1	1	0	1	1	1	1	1	1
9	0	0	0	0	0	1	1	0	1	1	0	1	1	1	1	1
10	1	0	0	0	0	1	1	0	0	1	0	0	0	1	0	1
11	0	1	1	1	0	1	1	0	0	1	0	0	0	1	0	1
12	0	0	0	0	0	0	0	0	0	1	0	0	0	0	0	1
13	0	0	0	0	1	0	0	0	0	1	0	0	0	0	0	1
14	0	0	0	0	1	0	0	0	0	0	1	0	0	1	0	1
15	0	0	0	0	1	0	0	0	1	0	1	0	1	1	1	1
16	0	0	0	0	1	0	0	1	1	0	1	0	1	1	1	1
17	0	0	0	0	0	0	1	1	1	0	1	1	1	1	1	1
18	0	0	0	0	0	1	1	0	1	1	0	1	1	1	1	1
19	1	0	0	0	0	1	1	0	0	1	0	0	0	1	0	1
20	0	1	1	1	0	1	1	0	0	1	0	0	0	1	0	1

The core oscillator and the extended model were implemented in a simple Android application that enables running the core oscillator of the cell cycle to see the activation/deactivation of the genes over time, and also the extended model with the phosphorylation events and watch how this is coordinated with the original model. Back to our suggestions, the user has the ability to apply different suggested effects and watch their impression on the model.

Finally, this work shed light on the existing gaps in our knowledge about the fission yeast, and pointed out the need for a comprehensive resource combining the information about regulatory effects of the genes whose expression levels oscillate during the cell cycle, and regulate or are regulated by the genes in the core oscillator.

## 4 Conclusion

Constructing and interpreting partial or full gene regulatory networks that describe the workings of cells is a very important aim of cell biology. Evidence for regulatory links may come from experiments carried out under different conditions, and these links may or may not be fully compatible to each other. Still, every step that expands and extends the current network may inspire other scientists to carry out subsequent experiments that may serve to either validate or falsify these hypothetical regulatory links. Eventually, if validated independently by several groups, the newly introduced regulatory links may become generally accepted.

In this work we extended the current cell cycle core oscillator of fission yeast by adding four genes whose products are being phosphorylated during the cell cycle. Since phosphorylation might lead to activation or deactivation of the substrate, our results suggest possible phosphorylation effects on the added gene products that can be validated or disproven in future experiments. Based on the Boolean network model presented by Davidich and Bornholdt, our results also suggest a possible mechanism to restart the cell cycle. Furthermore, we identified and discussed several unclear points regarding the mutual regulatory effects of the genes involved in the cell cycle.

We consider this work as a first step toward deriving an extended gene regulatory model of how the cell cycle oscillator of yeast couples to the rest of the cell. We argue that this may have important biotechnological implications. For example, there is significant current interest [30] in unraveling connections between the yeast cell cycle oscillator and metabolic oscillations. The cell cycle oscillator also appears to be important for “rerouting carbon fluxes [31] and to coordinate carbohydrate metabolism [32].

## Supporting information

S1 Fig The Android application use cases. (TIFF)

S1 Table Target genes and their ranks according to [2] [15] among the oscillating genes during cell cycle. The genes that do not oscillate are marked with # with their rank if their transcription levels slightly oscillate.
(XLSX)

S1 File Android application video tutorial. A short video tutorial on how to use the android application accompaying this work.
(MP4)

## References

[1] Nurse P. Universal control mechanism regulating onset of M-phase. Natur. 1990;344: 503–508. 10.1038/344503a0 10.1038/344503a0 2138713

[2] Bushel P, Heard N, Gutman R, Liu L, Peddada S, Pyne S. Dissecting the fission yeast regulatory network reveals phase-specific control elements of its life cycle. BMC Systems Biology. 2009;3(1):93 10.1186/1752-0509-3-93 19758441 10.1186/1752-0509-3-93 PMC2758837

[3] Simonetti F. Study of the mechanisms of sexual differentiation in the fission yeast S.pombe. PhD thesis, Universite Paris-Saclay, 2017. Available from: https://tel.archives-ouvertes.fr/tel-01513121/document

[4] Lim S, Kaldis P. Cdks, cyclins and CKIs: roles beyond cell cycle regulation. Development. 2013;140(15):: 3079–3093. 10.1242/dev.091744 23861057 10.1242/dev.091744

[5] Gérard C, Tyson J, Coudreuse D, Novák B. Cell Cycle Control by a Minimal Cdk Network. PLOS Computational Biology. 2015 6;11(2):, e1004056 10.1371/journal.pcbi.1004056 25658582 10.1371/journal.pcbi.1004056 PMC4319789

[6] Coudreuse D, Nurse P. Driving the cell cycle with a minimal CDK control network. Nature. 2010;468(7327):: 1074–1079. 10.1038/nature09543 21179163 10.1038/nature09543

[7] Garcia-Garcia T, Poncet S, Derouiche A, Shi L, Mijakovic I, Noirot-Gros M. Role of Protein Phosphorylation in the Regulation of Cell Cycle and DNA-Related Processes in Bacteria. Frontiers In Microbiology. 2016; 7 10.3389/fmicb.2016.00184 26909079 10.3389/fmicb.2016.00184 PMC4754617

[8] Swaffer M, Jones A, Flynn H, Snijders A, Nurse P. CDK Substrate Phosphorylation and Ordering the Cell Cycle. Cell. 2016;167(7): 1750–1761. 10.1016/j.cell.2016.11.034 27984725 10.1016/j.cell.2016.11.034 PMC5161751

[9] Hirose Y, Ohkuma Y. Phosphorylation of the C-terminal Domain of RNA Polymerase II Plays Central Roles in the Integrated Events of Eucaryotic Gene Expression. The Journal Of Biochemistry. 2007;141(5):601–608. 10.1093/jb/mvm090 17405796 10.1093/jb/mvm090

[10] Novak B, Tyson JJ. Modeling the control of DNA replication in fission yeast. Proceedings of the National Academy of Sciences. 1997;94(17):9147–9152. 10.1073/pnas.94.17.9147 10.1073/pnas.94.17.9147 PMC23080 9256450

[11] Davidich M, Bornholdt S. Boolean Network Model Predicts Cell Cycle Sequence of Fission Yeast. PLoS ONE. 2008;3(2):e1672 10.1371/journal.pone.0001672 18301750 10.1371/journal.pone.0001672 PMC2243020

[12] Davidich M, Bornholdt S. Boolean Network Model Predicts Knockout Mutant Phenotypes of Fission Yeast. PLoS ONE. 2013;8(9):e71786 10.1371/journal.pone.0071786 24069138 10.1371/journal.pone.0071786 PMC3777975

[13] Wood V, Harris M, McDowall M, Rutherford K, Vaughan B, Staines D et al Pombase: a comprehensive online resource for fission yeast. Nucleic Acids Research. 2011;40(D1):D695–D699. 10.1093/nar/gkr853 22039153 10.1093/nar/gkr853 PMC3245111

[14] Wilson D, Charoensawan V, Kummerfeld S, Teichmann S. Dbd taxonomically broad transcription factor predictions: new content and functionality. Nucleic Acids Research. 2007;36(suppl 1):D88–D92. 10.1093/nar/gkm964 18073188 10.1093/nar/gkm964 PMC2238844

[15] Oliva A, Rosebrock A, Ferrezuelo F, Pyne S, Chen H, Skiena S, Futcher B, Leatherwood J. The cell cycle regulated genes of schizosaccharomyces pombe. PLoS Biology. 2005;3(7):e225 10.1371/journal.pbio.0030225 15966770 10.1371/journal.pbio.0030225 PMC1157095

[16] Huang D, Sherman B, Lempicki R. Systematic and integrative analysis of large gene lists using david bioinformatics resources. Nature Protocols. 2009;4(1):44–57. 10.1038/nprot.2008.211 19131956 10.1038/nprot.2008.211

[17] Huang D, Sherman B, Lempicki R. Bioinformatics enrichment tools: paths toward the comprehensive functional analysis of large gene lists. Nucleic Acids Research. 2008;37(1):1–13. 10.1093/nar/gkn923 19033363 10.1093/nar/gkn923 PMC2615629

[18] Correa-Bordes J. p25rum1 promotes proteolysis of the mitotic B-cyclin p56cdc13 during G1 of the fission yeast cell cycle. The EMBO Journal. 1997;16(15):4657–4664. 10.1093/emboj/16.15.4657 9303310 10.1093/emboj/16.15.4657 PMC1170092

[19] Bandyopadhyay S, Dey I, Suresh M, Sundaram G. The Basic Leucine Zipper Domain Transcription Factor Atf1 Directly Controls Cdc13 Expression and Regulates Mitotic Entry Independently of Wee1 and Cdc25 in Schizosaccharomyces pombe. Eukaryotic Cell. 2014;13(6):813–821. 10.1128/EC.00059-14 24728197 10.1128/EC.00059-14 PMC4054271

[20] Benito J. Regulation of the G1 phase of the cell cycle by periodic stabilization and degradation of the p25rum1 CDK inhibitor. The EMBO Journal. 1998;17(2):482–497. 10.1093/emboj/17.2.482 9430640 10.1093/emboj/17.2.482 PMC1170399

[21] Mondesert O, McGowan C, Russell P. Cig2, a B-type cyclin, promotes the onset of S in Schizosaccharomyces pombe. Molecular And Cellular Biology. 1996;16(4):1527–1533. 10.1128/MCB.16.4.1527 8657126 10.1128/mcb.16.4.1527 PMC231137

[22] Ng S, Anderson M, White S, McInerny C. mik1+G1-S transcription regulates mitotic entry in fission yeast. FEBS Letters. 2001;503(2-3):131–134. 10.1016/S0014-5793(01)02720-X 11513869 10.1016/s0014-5793(01)02720-x

[23] Simanis V, Nurse P. Characterization of the fission yeast cdc10 protein that is required for commitment to the cell cycle. J. Cell Sci. 1989;92:51–56. 2777915 10.1242/jcs.92.1.51

[24] Tyson J, Csikasz-Nagy A, Novak B. The dynamics of cell cycle regulation. Bioessays. 2002;24(12):1095–1109. 10.1002/bies.10191 12447975 10.1002/bies.10191

[25] Rustici G, Mata J, Kivinen K, Li P, Penkett C, Burns G, Hayles J, Brazma A, Nurse P, Bhler J. Periodic gene expression program of the fission yeast cell cycle. Nature Genetics. 2004;36(8):809–817. 10.1038/ng1377 15195092 10.1038/ng1377

[26] Shimada M, Yamada-Namikawa C, Murakami-Tonami Y, Yoshida T, Nakanishi M, Urano T, Murakami H. Cdc2p controls the forkhead transcription factor Fkh2p by phosphorylation during sexual differentiation in fission yeast. The EMBO Journal. 2007;27(1):132–142. 10.1038/sj.emboj.7601949 18059475 10.1038/sj.emboj.7601949 PMC2206131

[27] Shiozaki K, Russell P. Conjugation, meiosis, and the osmotic stress response are regulated by Spc1 kinase through Atf1 transcription factor in fission yeast. Genes & Development. 1996;10(18):2276–2288. 10.1101/gad.10.18.2276 8824587 10.1101/gad.10.18.2276

[28] Sveiczer A, Csikasz-Nagy A, Gyorffy B, Tyson J, Novak B. Modeling the fission yeast cell cycle: Quantized cycle times in wee1- cdc25Delta mutant cells. Proceedings Of The National Academy Of Sciences. 2000;97(14):7865–7870. 10.1073/pnas.97.14.7865 10.1073/pnas.97.14.7865 PMC16636 10884416

[29] Tanaka K, Okayama H. A Pcl-like Cyclin Activates the Res2p-Cdc10p Cell Cycle “Start” Transcriptional Factor Complex in Fission Yeast. Molecular Biology Of The Cell. 2000;11(9):2845–2862. 10.1091/mbc.11.9.2845 10982385 10.1091/mbc.11.9.2845 PMC14960

[30] Morgan L, Moses G, Young RM. Coupling of the cell cycle and metabolism in yeast cell-cycle-related oscillations via resource criticality and checkpoint gating. Letters in Biomathematics. 2018;5(1):, 113–128. 10.1080/23737867.2018.1456366

[31] Ewald JC, Kuehne A, Zamboni N, Skotheim JM. The Yeast Cyclin-Dependent Kinase Routes Carbon Fluxes to Fuel Cell Cycle Progression. Mol Cell. 2016;62(4):, 532–545. 10.1016/j.molcel.2016.02.017 27203178 10.1016/j.molcel.2016.02.017 PMC4875507

[32] Zhao G, Chen Y, Carey L, Futcher B. Cyclin-Dependent Kinase Co-Ordinates Carbohydrate Metabolism and Cell Cycle in S. cerevisiae. Mol Cell. 2016;62(4):, 546–557. 10.1016/j.molcel.2016.04.026 27203179 10.1016/j.molcel.2016.04.026 PMC4905568

