# Supplementary figures and images for: Adding phosphorylation events to the core oscillator driving the cell cycle of fission yeast
Source: PLoS One. 2018 Dec 4;13(12):e0208515. doi: 10.1371/journal.pone.0208515 (PMC6279014; doi:10.1371/journal.pone.0208515)

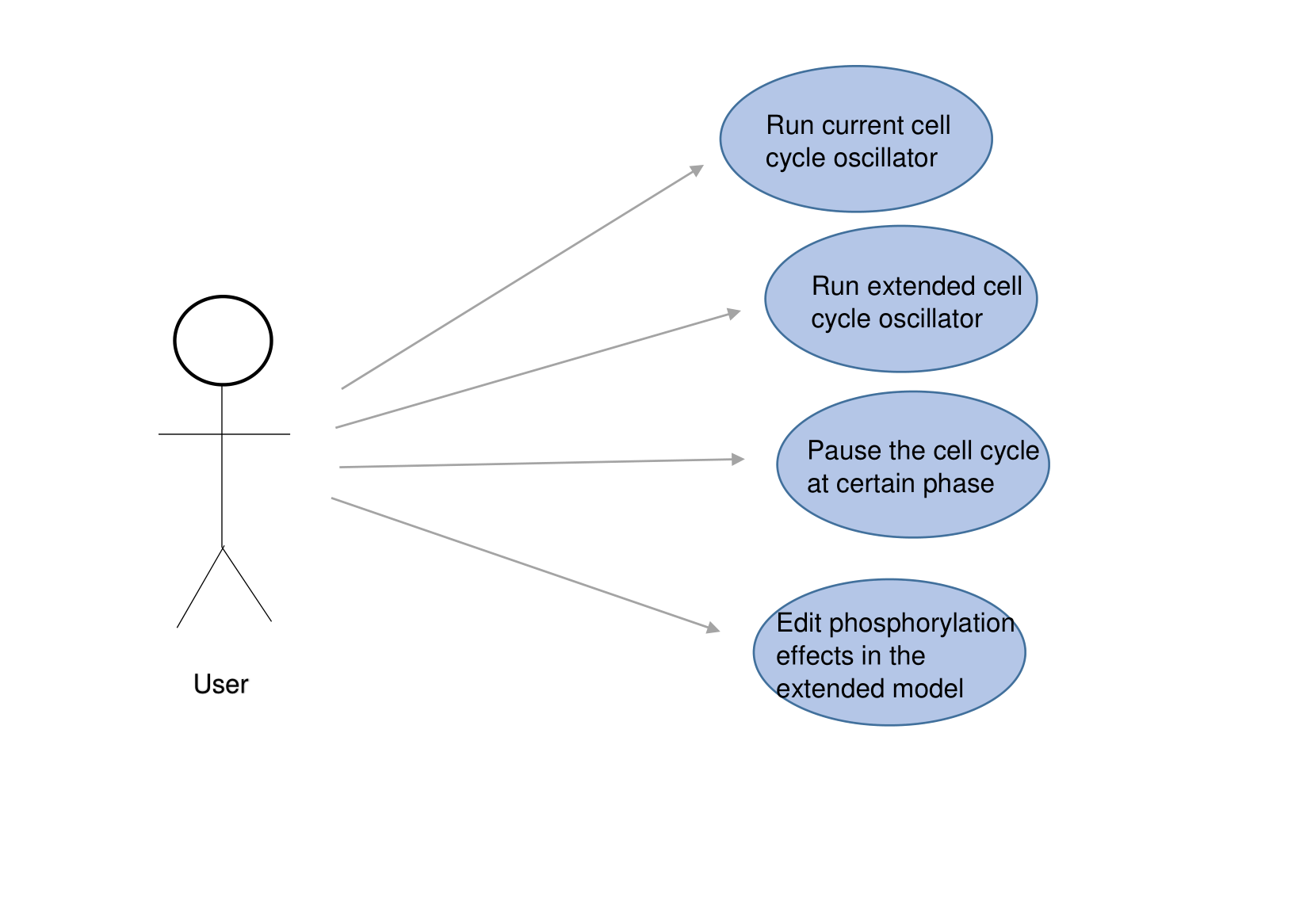

Supplement: S1 Fig — (TIFF) [file pone.0208515.s001.tiff]
